# Age-associated chromatin relaxation is enhanced in Huntington's disease mice

**DOI:** 10.18632/aging.101193

**Published:** 2017-03-12

**Authors:** Myungsun Park, Byungkuk Min, Kyuheum Jeon, Sunwha Cho, Jung Sun Park, Jisun Kim, Jeha Jeon, Jinhoi Song, Seokho Kim, Sangkyun Jeong, Hyemyung Seo, Yong-Kook Kang

**Affiliations:** ^1^ Development and Differentiation Research Center, KRIBB, Yuseong-gu, Daejeon, 305-806, South Korea; ^2^ Aging Research Institute, Korea Research Institute of Bioscience and Biotechnology, Daejeon, 305-806, South Korea; ^3^ Department of Functional Genomics, University of Science and Technology (UST), Yuseong-gu, Daejeon, 305-350, South Korea; ^4^ Mibyeong Research Center, Korea Institute of Oriental Medicine (KIOM), Yuseong-gu, Daejeon, 305-811, South Korea; ^5^ Department of Molecular and Life Sciences, Hanyang University, Sangnok-gu, Ansan, Gyeonggi-do, 15588, South Korea

**Keywords:** aging, Huntington's disease, targeted NGS, epigenetic, chromatin accessibility, Huntingtin (HTT)

## Abstract

Expansion of polyglutamine stretch in the huntingtin (HTT) protein is a major cause of Huntington's disease (HD). The polyglutamine part in HTT interacts with various proteins implicated in epigenetic regulation of genes, suggesting that mutant HTT may disturb the integrity of the epigenetic system. Here, we used a PCRseq-based method to examine expression profile of 395 exonic segments from 260 “epi-driver” genes in splenic T lymphocytes from aged HD mice. We identified 67 exonic segments differentially expressed between young and aged HD mice, most of them upregulated in the aged. Polycomb-repressive complex (PRC)-regulated genes (PRGs) were markedly upregulated in aged HD mice, consistent with downregulation of PRC genes. Epi-driver gene categories of lysine-methylation, lysine-demethylation, arginine-methylation, and PRG showed differential age-associated changes between HD and control. Analyzing the pattern of change in epi-driver gene expressions hinted at an enhanced shift in HD chromatin to a more accessible state with age, which was experimentally demonstrated by DNase-I-hypersensitivity sequencing showing increased chromatin accessibility in HD cells compared to control. We suggest the global change can potentially relieve chromatin-induced repression of many genes, and the unintended expressions of some detrimental proteins could alter T cell function to a greater degree in aged HD mice.

## INTRODUCTION

Huntington's disease (HD) is caused by a mutation that leads to an expansion of a polyglutamine stretch in the huntingtin (HTT) protein. Disease pathology has been traditionally confined to the central nervous system; however, mutated huntingtin (mHTT) is ubiquitously expressed throughout the body in humans and other mammals [1-4]. HD is associated with abnormalities in peripheral tissues and results in various symptoms, including weight loss, skeletal muscle atrophy, cardiac failure, testicular atrophy, osteoporosis, and blood cell dysfunction, none of which are considered connected to neurological dysfunction or general sickness (summarized in [5]).

Blood cells are affected in many ways in HD, with HD hematic cells exhibiting altered gene transcription [6, 7], caspase activity [8], and mitochondrial activity [9, 10]. Immune-system dysfunction is a noticeable symptom of HD pathogenesis in the periphery, where characteristic increase in the levels of circulating pro-inflammatory cytokines [11] and chemokines [12] are observed in the blood many years prior to disease onset. Additionally, HD myeloid cells are hyperactive in response to stimulation [13] and exhibit functional deficits when migrating to chemotactic stimuli [14] and phagocytosing fluorescent beads [15]. Furthermore, the level of mHTT in T lymphocytes was correlated with disease-burden scores and caudate atrophy [16]. These collective findings suggest that mHTT expression causatively affects immune-system integrity and T lymphocyte function.

The precise function of HTT is unclear. HTT is implicated in several cellular processes, including transcriptional regulation. Wild-type (wt) HTT is known to interact with a number of transcription factors and regulators, such as the tumor suppressor TP53 [16], NF-κB [17], CREB-binding protein (CBP) [16], NeuroD [18], SP1 [19], CA150 [20], TAFII130 [19], NCOR [21], CtBP [22], and REST/NRSF [23], to promote and/or repress gene transcription. Because these HTT partners are ubiquitously expressed, mHTT may disturb transcriptional mechanisms common to many genes [24, 25] and hamper gene transcription in various peripheral tissues, although the mechanism of affecting the activity of transcriptional factors in HD is quite complex including inhibition, sequestration, protein degradation, and transcriptional dysregulation. Substantial alterations in mRNA expression were reported in various tissues of HD transgenic mice [26-28] and in the blood of HD patients [6, 29-31]. Notably, many of HTT partners are chromatin modifiers and remodelers involved in epigenetic regulation, which raises the possibility that the integrity of the epigenetic system is affected in mHTT-expressing cells.

The relevance of epigenetics in human disease has been extended from the field of cancer research to several diverse conditions, including a variety of neuropathologies. HD has emerged as a prototypical paradigm of epigenetic dysregulation in a neurodegenerative condition [32]. Two lines of discovery have placed epigenetics central to neurodegenerative fields: reports of therapeutic benefits associated with histone deacetylase (HDAC)-inhibitor drugs in HD and genetic findings linking neuropathy or cerebellar ataxia-associated adult-onset dementia to deleterious mutations in proteins implicated in regulation of DNA methylation and covalent histone modifications [33]. These seminal observations led to the general hypothesis of epigenetic imbalance as an important characteristic in neurodegeneration. Both transcriptional and epigenetic dysregulation in HD models are important and early molecular events in the pathology [32]; however, it is unclear to what causative extent altered epigenetics plays in the altered transcriptional program observed in HD.

In this study, we examined the expression profile of “epi-driver” genes that encode epigenetic players functioning as writers, readers, and erasers of chromatin marks in the nucleus of splenic T lymphocytes from aged HD mice. We included several hundreds of epi-driver genes and their related genes in the analysis and measured their expression levels by group (functional category) using the spiking-in a neighbor genome for competitive PCR-amplicon sequencing (SiNG-PCRseq) method [34]. SiNG-PCRseq uses a genome of closely related species to the sample, i.e. neighbor genome, as an array of competitor templates to exploit genome-wide chemically equivalent but easily discriminable homologous sequences with a known copy arrangement in it. Multiplexed competitive PCRs using mouse cDNAs with a spike-in of the rat genome and subsequent high-throughput sequencing let us acquire very accurate relative quantities of murine epi-driver cDNA sequences to those of matched rat genomic DNA sequences. These relative quantities of different cDNA sequences are, as we know the quantitative relationship between different competitor templates in the neighbor genome, in turn comparable to one another in a given sample as well as between different samples after simple normalization. This large-scale analysis is necessary, especially given the strong functional redundancy among family member genes and the necessity of the relative expression of all redundant genes to be measured at the same time and under the same conditions. Our efforts could, we hope, provide insight into the complexity of HD-related epigenetics, HD pathogenesis and to the role of epigenetic dysregulation in other neurodegenerative diseases.

## RESULTS

### Gene expression analysis of young and aged HD mice using SiNG-PCR sequencing

**Figure 1:**
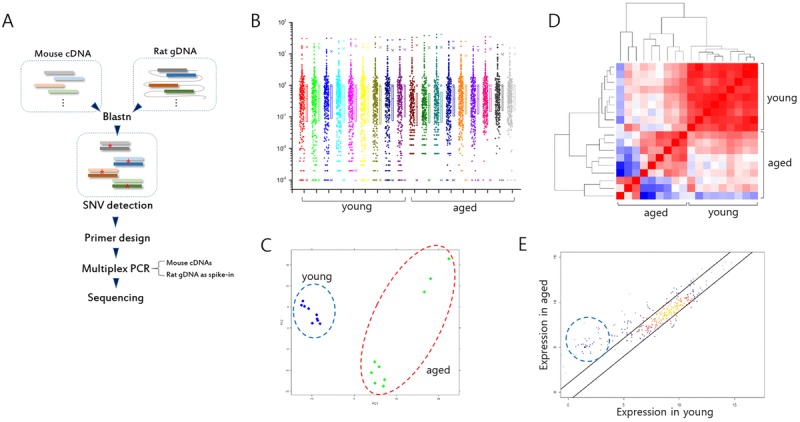
Analysis of gene expression in splenic T cells from mouse models of Huntington's disease (HD) (**A**) Illustration of spiking-in neighbor genome PCR (SiNG-PCR) sequencing. (**B**) Distribution of relative counts of target-gene amplicons in young and aged HD mice. The ratio (M/R) of cDNA counts relative to rat spike-in counts was calculated to measure the expression level of each target amplicon. (**C**) Principal component analysis (PCA). Both young and aged sample groups are marked using colored circles. (**D**) Heatmap illustrating Pearson correlation between young and aged group samples. (**E**) Scatter plot (*r* = 0.9). Those of amplicons whose negligible expressions in the young group are markedly enhanced in the aged group are circled.

For expression analysis using multiplex-PCR, we selected a variety of epi-driver genes directly involved in epigenome modification, such as histone modifiers, DNA methylases, and other chromatin-structure regulators. Additionally, we also included senescence-associated genes and developmental genes regulated by Polycomb group (PcG) proteins; they were selected based on our own research interests and gleaned from a variety of recent research papers. The epi-driver gene panel consisted of 395 exonic amplicon sequences derived from 260 genes in total. We analyzed T lymphocytes from the spleens of young (2-month old) and aged (16–19-month old) HD mice [FVB-Tg(YAC128)53Hay/J] carrying the human *HTT* gene containing 128 CAG trinucleotide repeats. Endogenous mouse *Htt* was similarly expressed in wt and HD T cells as shown previously [16], and also between young and aged HD samples (Supplementary Fig. S1). The human *HTT* transgene, which is driven by its own promoter, was expressed only in HD mice at a level comparable to that of endogenous *Htt* (Supplementary Fig. S1). Using rat genomic DNA as a spike-in, spike-in neighbor genome-PCR (SiNG-PCR) [34] was performed, and the resulting amplicons were processed for Illumina sequencing, as illustrated in Figure 1A. The sequences of all competing amplicons were the same except single or few nucleotide positions carrying species-specific variations that could be discriminated after amplicon sequencing. In total, an average of 8.8 × 10^6^ reads were obtained from each sample, 62.3% of which mapped exactly to our amplicon-built reference.

**Figure 2:**
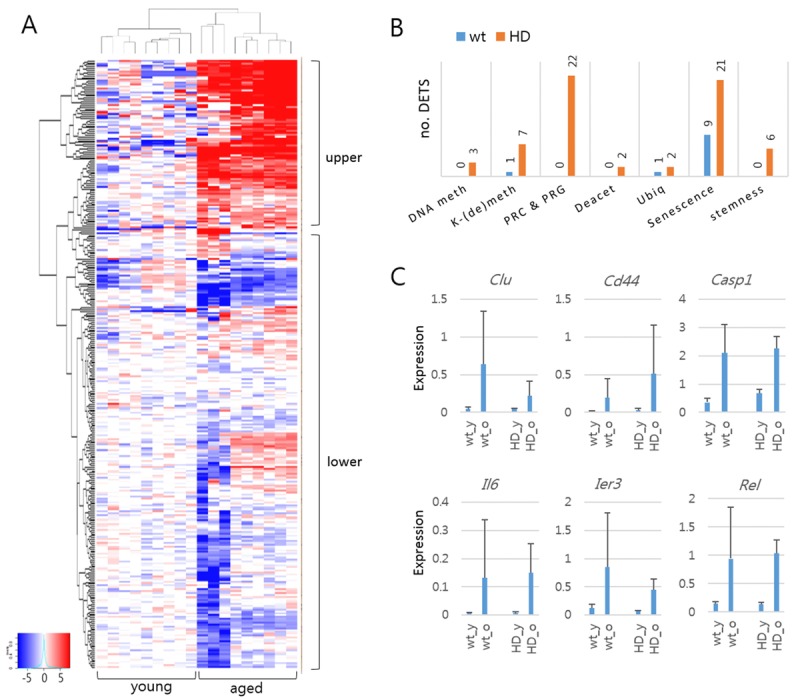
Differentially expressed target sequences (DETSs) in young and aged mouse models of Huntington's disease (HD) (**A**) Heatmap of amplicon levels. The heatmap is arbitrarily divided into upper and lower areas, with the former containing highly expressed amplicons from the aged samples. (**B**) Comparison of DETSs in different functional categories between wild-type and HD samples. The number of DETSs in each category is indicated on the bar. DNA meth, DNA methylation; K-(de)meth, lysine methylation and demethylation; PRC & PRG, Polycomb group proteins and PCR-regulated genes; Deacet, histone deacetylation; Ubiq, ubiquitination. (**C**) Expression levels of senescence-category DETSs commonly detected in wild-type and HD samples. Error bars, standard deviation.

The expression level of each exonic sequence was measured by calculating the M/R ratio, the ratio of the mouse read counts relative to the rat's counts. The distribution of M/R values for every target sequence is shown in Figure 1B. Principal component analysis (PCA) using the normalized M/R inputs showed differences between the young and the aged HD mice (Fig. 1C), and also between the aged wt and the aged HD mice (Supplementary Fig. S2). For aged HD samples, 16 month- and 19 month-old mice were used, and the three strong outliers in the PCA plot (also see Fig. 2A) were obtained from the older whereas the others were from the younger. We speculate that the transcriptomic distinction may come from the age difference between the aged HD samples. Unsupervised correlation analysis showed that within-group correlation was significantly stronger in the young HD than the aged HD mice group (Fig. 1D and see also Fig. S2), indicating weakening of expression control in the latter group. In the scatter plot, those of genes that were minimally expressed (M/R < 0.01) in the young HD mice showed a noticeable increase in expression level in the aged HD mice (Fig. 1E), suggesting possible derepression of normally repressed genes during the course of aging (see below).

### Identification of differentially expressed target sequences between young and aged HD mice

Figure 2A shows the heat map of amplicon levels between young and aged HD mice. Using DESeq2 (http://bioconductor.org/packages/release/bioc/html/DESeq2.html), we identified 67 differentially expressed target sequences [DETS; fold change > 2; false discovery rate (FDR) < 0.01] in the HD mice, most of which were upregulated in the aged HD mice (Table 1). These DETSs outnumbered the DETSs that identified in the comparison of wt young and aged mice by five to one (Supplementary Fig. S3). We classified the amplicons by function of their corresponding genes and found that many of the DETSs belonged to the senescence category [31% (21/67) and 64% (9/14) in HD and wt samples, respectively] (Fig. 2B). Of the 14 DETSs found in wt samples, nine were also detected as HD DETSs, with eight belonging to the senescence category (*Clu*, *Cd44*, *Casp1*, *Il6*, *Rel*, and *Ier3*) (Fig. 2C). These results showed the ability of the gene-expression analysis system to pinpoint previously identified differences between samples. In addition to the senescence-category DETSs, there were Polycomb-repressive complex (PRC)- and PRC-regulated gene (PRG)-associated DETSs that occupied the largest proportion [32% (22/67)] among the DETSs in HD mice (Fig. 2B).

**Table 1:** Differentially expressed exonic sequences in splenic T cells between young and aged HD mice

amp_id*	Category	log_2_FC*	FDR***	amp_id	Category	log_2_FC	FDR
***Cd44_1***	Aging	4.146	1.03E-12	***Scmh2_1***	PRC	2.261	0.00071504
***Rel_26***	Aging	2.908	2.12E-12	***Nanog_1***	Stemness	4.108	0.00073542
***Ier3_2***	Aging	2.534	7.69E-09	***Setd2_1***	K-meth	1.480	0.000769123
***Hdac10_1***	Deacet	2.250	5.03E-08	***Lmx1b_1***	PRG	3.993	0.000894789
***Il6_2***	Aging	4.598	0.000000229	***Clu_1***	Aging	2.441	0.001019842
***Eif2c4_1***	RNAi	4.141	0.000000266	***Casp1_1***	Aging	1.714	0.001462471
***Il6_1***	Aging	4.493	0.00000034	***Clu_2***	Aging	2.287	0.001692421
***Ier3_1***	Aging	2.740	0.000000707	***Pax7_2***	PRG	3.651	0.00266687
***Zic2_2***	PRG	4.234	0.00000115	***Rnf39_1***	Ubiq	3.159	0.00266687
***Cbx5_1***	PRC	−2.156	0.00000352	***Prmt5_3***	Arg_meth	−1.253	0.003176115
***Ig1_108***	Aging	4.266	0.00000357	***Lbr_1***	Chr_str	−1.496	0.003415818
***Uhrf1_2***	DNA_meth	−2.049	0.00000388	***Onecut1_6***	PRG	3.537	0.003788077
***Zic2_1***	PRG	3.303	0.00000389	***Pik3cd_9***	Aging	−1.281	0.004038879
***Cbx5_3***	PRC	−2.145	0.00000487	***Tbx3_3***	PRG	2.982	0.004288935
***Cbx3_3***	PRC	5.257	0.00000524	***Uhrf1_4***	DNA_meth	1.754	0.004416562
***Hdacc9_2***	Deacet	3.309	0.00000539	***Kdm6b_3***	K-demeth	1.058	0.004532551
***Tbx3_4***	PRG	2.744	0.0000242	***Slc14a1_1***	Aging	−1.036	0.004691194
***Rnf39_2***	Ubiq	3.898	0.0000711	***Otx2_1***	PRG	4.790	0.004691194
***Cdx2_1***	PRG	4.403	0.000072	***Ppargc1a_1***	Aging	3.435	0.004965943
***Axin2_1***	Stemness	−1.727	0.000072	***Prdm2_3***	K-meth	2.067	0.004965943
***Jmjd7_1***	K-demeth	2.028	0.0000773	***Hoxa1_1***	PRG	3.181	0.004965943
***Bub1b_51***	Aging	−1.540	0.000139024	***Hoxb1_3***	PRG	3.249	0.004965943
***Slc14a1_2***	Aging	−1.644	0.000145455	***Ppargc1a_1***	Aging	3.280	0.005252247
***Kdm6b_2***	K-demeth	1.500	0.000145455	***Chaf1a_3***	chaperon	−0.998	0.005664136
***Casp1_2***	Aging	1.738	0.000154164	***Prdm9_4***	K-meth	2.446	0.005664136
***Ppargc1a_2***	Aging	4.100	0.00018008	***Neurog1_1***	PRG	3.446	0.006245221
***Sox6_3***	PRG	1.976	0.000209766	***Pax7_3***	PRG	3.786	0.006245221
***Nkx2.2_3***	PRG	4.032	0.000328876	***Rbl2_2***	Stemness	−1.331	0.006245221
***Deptor_46***	Aging	2.549	0.000334226	***Nodal_2***	Stemness	3.430	0.006294356
***Dnmt1_2***	DNA_meth	−1.651	0.00045605	***Nkx2.2_2***	PRG	3.503	0.007498966
***Phc3_4***	PRC	1.980	0.000664392	***Ash1l_7***	K-meth	1.597	0.008258801

* indicated by gene name

** Expression levels of aged over young samples. FC, fold-change

*** FDR, false discovery rate

### Target genes of the Polycomb-repressive complex were significantly upregulated in aged HD mice

**Figure 3:**
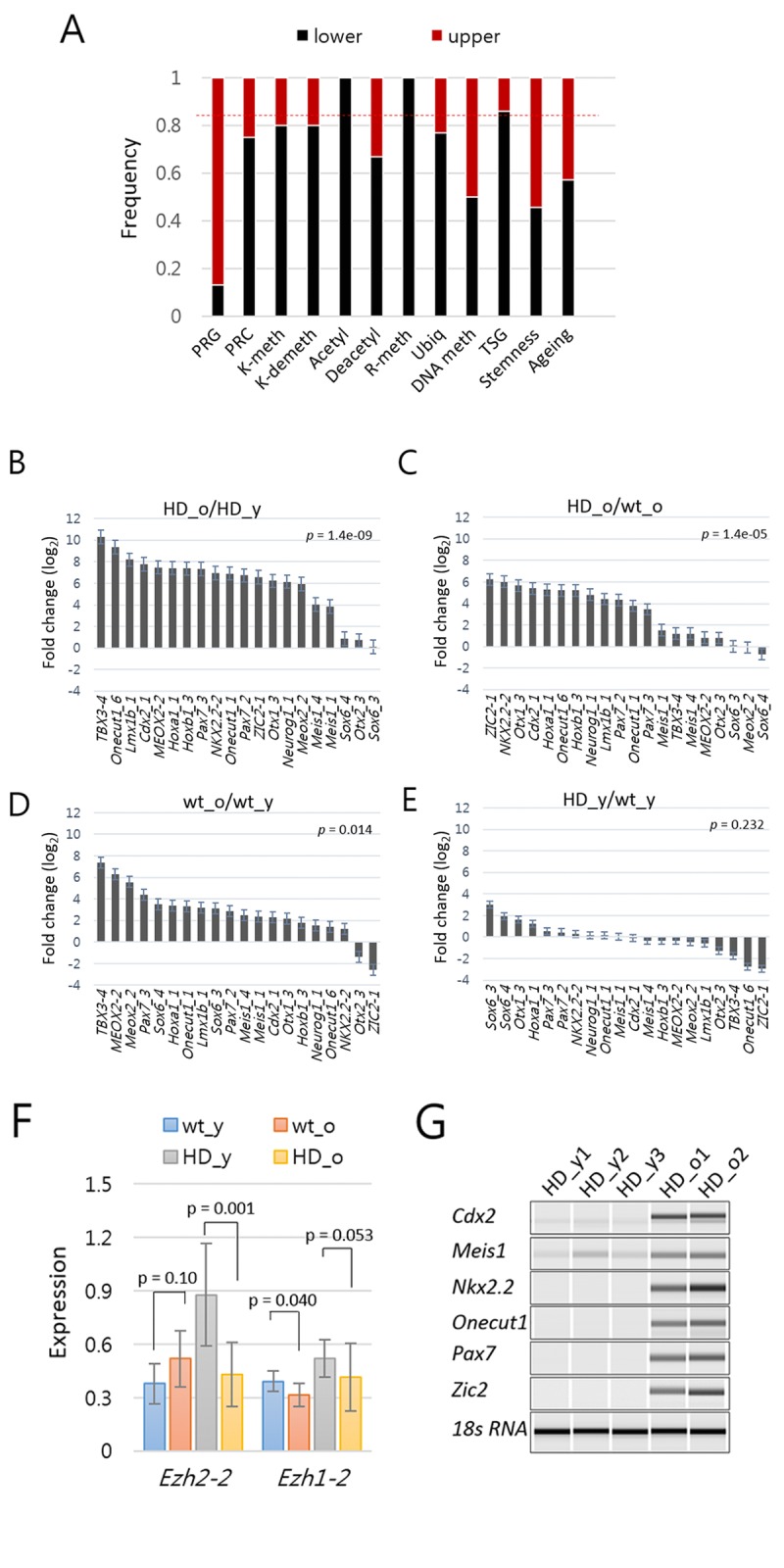
Increased expression of Polycomb repressive complex (PRC)-regulated genes (PRG) in aged mouse models of Huntington's disease (HD) (**A**) The proportion of amplicons in each category that belong to the upper (red) and lower (black) clusters in the heat map shown in Figure 2A. The upper cluster amplicons represent 26% of the total amplicons as indicated by dotted line (red). TSG, tumor suppressor genes. For other abbreviations, refer to the Figure 2 legend. (**B-E**) Fold difference (log_2_) in the amount of PRG amplicons between aged HD *vs*. young HD (**B**), between aged HD *vs*. aged wild-type (wt) (**C**), between aged wt and young wt (**D**), and between young HD *vs*. young wt samples (**E**). *P*-values, paired-sample *t*-test. Error bar, standard error. (**F**) Comparison of expression levels of *Ezh2* in HD and wild type samples. *P*-value, paired-sample *t*-test. Error bar, standard deviation. (**G**) RT-PCR analysis of PRGs. PRGs were randomly chosen, and expression levels were examined in splenic T cells from young (HD_y) and aged HD (HD_o) mice. 18s RNA, loading control.

The heat map in Figure 2A shows two large upper (86 amplicons; 26%) and lower (242 amplicons; 74%) clusters, with the upper one consisting of genes highly expressed in the aged HD mice. Profiling the functional categories associated with the aged-high amplicons revealed that the cluster was enriched with PRGs. Of the 23 PRGs in the panel, 19 (83%) were present in this cluster (Fig. 3A). Although the transcript levels of PRGs were very low (see below), they were consistently high in aged HD mice compared with those in young HD (Fig. 3B) and also compared with those in aged wt mice (Fig. 3C). Similar findings were observed in wt mice, although the aged-to-young difference was smaller (Fig. 3D). In the comparison between young HD and wt samples, there was no biased expressions of PRGs (Fig. 3E). In line with the increased expression of PRGs, *Ezh2*, the H3K27-methylating enzyme that activates PRC2-mediated chromatin repression was downregulated in aged HD mice (Fig. 3F). This result was reminiscent of previous reports that Ezh2 deficiency and subsequent decrease of repression-associated H3K27me3 lead to a rapid senescence [35, 36]. Our result suggested that the age-dependent derepression of PRGs was biased toward HD mice. Wt HTT interacts with Ezh2 to facilitate the histone H3K27 trimethylase activity of PRC2 [37]. It remains unclear whether mHTT can maintain interaction with Ezh2; however, our result suggested that mHTT dysregulated *Ezh2* at the mRNA level. We verified the derepression of various PRGs in aged HD mice by RT-PCR (Fig. 3G).

### Analysis of the pattern of age-associated change in epi-driver gene expression suggests a shift in chromatin to a more relaxed and accessible state in HD mice

**Figure 4:**
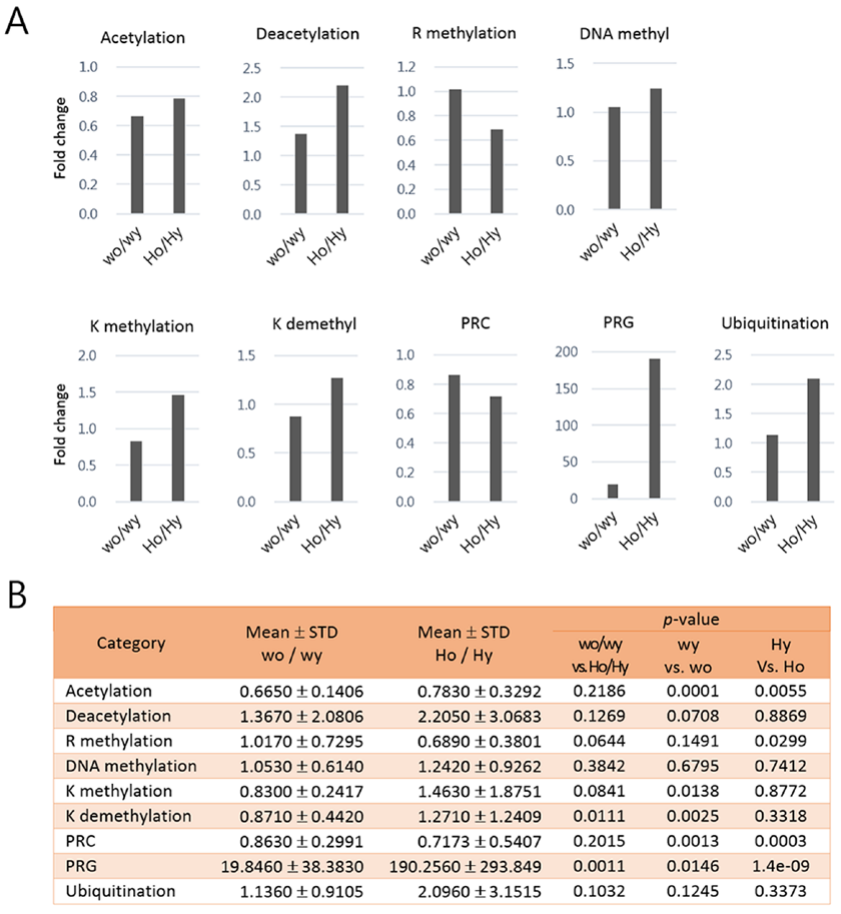
Comparison of the mean ratios of epi-driver gene expression levels in each category between young and aged samples of Huntington's disease (HD) mice and control mice In (**A**) each bar shows the fold change of epi-driver gene expression levels in each category between aged and young samples. In (**B**) the mean fold changes, standard deviations (STD), and p-values (paired-sample *t*-test) between indicated comparisons are represented for the graphs in A. Wo and wy, old and young wild-type samples; Ho and Hy, old and young HD samples.

We next calculated mean ratios of the expression levels of epi-driver genes in each category between aged and young samples in HD and wt control mice (Fig. 4). Acetylation-category transcripts were downregulated with great consistency in the aged samples in both wt (*p* = 0.0001) and HD (*p* = 0.0055) groups while there was no age-related difference (*p* = 0.2186) between the HD and wt groups. K methylation- and K demethylation-category genes were significantly downregulated in the aged wt samples whereas they appeared to be oppositely upregulated (though not significantly) in the aged HD samples. PRC-category genes showed age-related expression difference in the HD samples (*p* = 0.0003) and to a lesser extent in the wt samples (*p* = 0.0013). PRG-category amplicons exhibited age-associated difference in both wt and HD samples but the difference was far more significant in the HD samples (*p* = 1.4e-09 vs. *p* = 0.0146; see also Fig. 3) and, more importantly, they showed the highest age-associated difference (*p* = 0.0011) between the HD and wt mice. The expression levels of R methylation- category genes were also altered in the aged HD (*p* = 0.0299).

**Figure 5:**
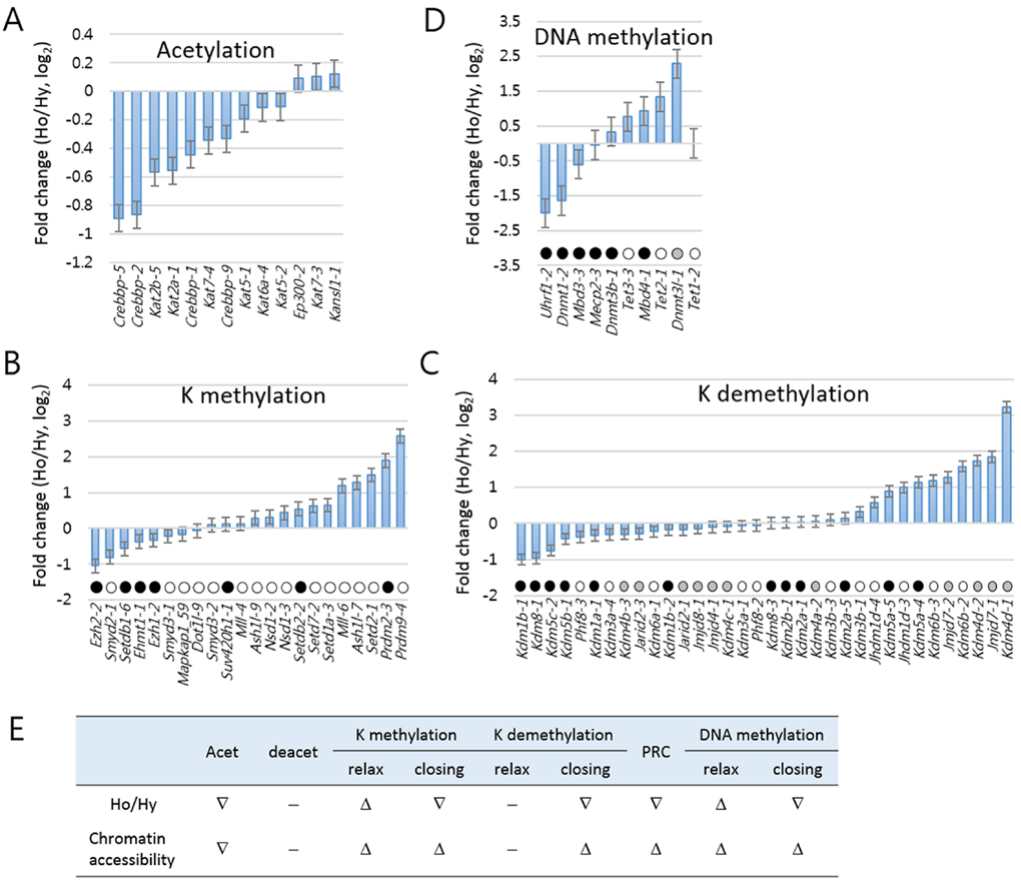
Analysis of age-associated pattern of change in epi-driver gene expressions in Huntington's disease mice Fold changes were measured for the epi-driver gene amplicons in the categories of acetylation (**A**), lysine (K) methylation (**B**), K demethylation (**C**), Polycomb-repressive complex (PRC, **C**), and DNA methylation (**D**). In **B-D**, amplicons are differentially marked according to their modification effects on chromatin accessibility: open circles indicate increased accessibility; solid circles indicate reduced accessibility; and grey circles for cases involving either increased or reduced accessibility. (**E**) Summary of the upward (D) or downward (∇) change in expression of epi-driver genes in each category. ‘-’ indicate no significant change. Ho and Hy, old and young HD samples.

We measured the fold changes in the amount of individual amplicons between the aged and the young samples of HD mice (Fig. 5). The acetylation-category amplicons were mostly downregulated in aged HD samples (Fig. 5A, see also Fig. 4). A variety of genes are involved in histone K-methylation and demethylation. Lysine methyltransferases can switch chromatin to a relaxed or closed state depending upon which lysine residues they attack, similar to lysine demethylases. In the aged HD mice, chromatin-relaxing lysine methyltransferases, such as *Setd2* (H3K36-specific), *Ash1l* (H3K4), and *Prdm9* (H3K4), were upregulated whereas chromatin-packing enzymes, such as *Ezh2* (H3K27) and *Setdb1* (H3K9), were downregulated (Fig. 5B). Among the lysine demethylases, *Kdm4d* (H3K9-specific) and *Kdm6b* (H3K27) were upregulated, whereas *Kdm1b* (H3K4), *Kdm5c* (H3K4), and *Kdm8* (H3K36) were downregulated in the aged HD mice (Fig. 5C). We also examined the K methylation- and K demethylation-category genes in the wt samples but they did not exhibit the pattern of change observed in the HD samples (Supplementary Fig. S4). We already identified the underrepresentation of PRC genes in the aged HD mice (Fig. 4), which agreed with the upregulation of PRGs in the same set of samples (Fig. 3 and 4). In DNA-methylation category, *Dnmt1* and *Uhrf1,* both involved in the maintenance of methylation, were downregulated in the aged HD samples (also see Supplementary Fig. S5), whereas *Tet2* and *Tet3*, which are implicated in DNA demethylation, were upregulated (Fig. 5D). Figure 5E summarizes the upward or downward changes in expression levels of epi-driver genes in each category according to HD mouse age. Most of the changes are evident of relaxed chromatin structure and increased chromatin accessibility to nuclear proteins. These changes are, however, ambiguous in the wt samples (Supplementary Fig. S4) and thus considered to be characteristic of HD samples.

### DNase-I hypersensitivity sequencing (DNase-seq) result demonstrates an increase of chromatin accessibility in aged HD cells compared to wild-type control

**Figure 6:**
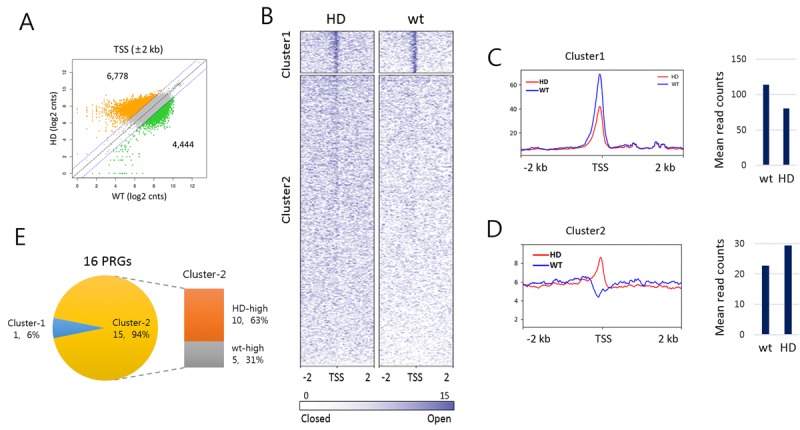
DNase-I hypersensitive site profiling in aged Huntington's disease (HD) and wild-type splenic cells (**A**) Scatter plot of DNase-I hypersensitive (DHS) read counts (log_2_) between HD and wt around transcription start site (TSS). DHS reads ± 2 kb around TSSs were counted. Colored dots (orange for HD-high and green for wt-high) indicate DHSs reads with 2-fold or more count differences. (**B**) Heatmaps of DHS signals around TSSs (± 2 kb) in HD and wt mice. DHS signals were clustered by the *k*-means algorithm (*k* = 2). Sites are ordered by DHS signal intensity around TSS. (**C-D**) Read count distribution around TSSs (± 2 kb, left) and the mean DHS signal density around TSSs (± 300 bp, right) in cluster-1 (C) and cluster-2 (D). (**E**) Comparison of DHS signal density at PRG TSSs between HD and wt samples. Shown are the proportions of PRGs with HD-high (orange) and wt-high (gray) DHS signals. Of the 16 PRGs, 15 PRGs belong to cluster-2 and 10 (63%) have denser signals in HD sample than in wt.

To examine if there was alteration in chromatin accessibility at transcription start site (TSS) in aged HD samples, we performed DNase I hypersensitivity (DHS) assay using splenic mononuclear cells obtained from 15 −16 month-old HD mice. Fifty - 100 bp in size of DNase I-digested genomic DNA fragments that were largely derived from nucleosome-deficient genomic regions [40] were collected and sequenced. We inspected DHS signals at 36,728 TSSs (± 2 kb) in aged HD and wt samples. As shown by a scatter plot in Fig. 6A (*r* = 0.32), DHS signals were shown skewed toward HD sample as they were enriched in HD sample more than in wt (6,778 vs. 4,444 TSSs, respectively). A clustering analysis using the *k*-means algorithm divided the 36,728 TSSs in total into two clusters (Fig. 6B). Cluster-1 consisted of 4,994 gene promoters having relatively condensed DHS reads at TSSs in both HD and wt samples, with the average read density marginally smaller in HD sample (fold-change = 0.704, *p* < 2.2e-16; Fig. 6C). On the contrary, the remained 31,734 gene promoters in cluster-2 displayed rather shallow DHS signals at TSSs, and these weak signals were more enriched in HD sample compared to wt (fold-change = 1.287, *p* < 2.2e-16; Fig. 6D). We examined the 16 PRGs listed in Fig. 3. All of them except *Meis1* belonged to the cluster-2 and two-third (10 PRGs) of them had higher DHS read counts in HD sample, agreeing with the upregulation of PRGs in aged HD sample (Fig. 3C). The enrichment of weak DHS signals in cluster-2 indicates that HD chromatin becomes more accessible than wt chromatin with age, conforming to the theory of an enhanced shift in chromatin to a more relaxed structure with age in HD sample. Together, it suggests that age-associated deregulation of overall chromatin structure increases decondensation of closed chromatin, which subsequently results in a partial activation of normally repressed genes.

## DISCUSSION

Our results from the expression analysis of epi-driver genes and DNase-seq experiment indicated that aging was accompanied by chromatin-conformation changes, shifting chromatin into a more relaxed and accessible state. HTT mutation appears to speed up and/or broaden the epigenomic alterations, and the resulting global changes subsequently causes de-repression of many genes that should otherwise have been kept tightly suppressed. Concomitantly increasing transcriptional noise over the aging genome and accompanying chaotic biochemical activities of proteins derived from leaked mRNAs can disturb homeostasis in cell physiology. The reduced expression of epi-driver genes implicated in chromatin-mediated gene repression may be responsible for the activation of silenced genes, possibly through repositioning insufficient transcriptional repressors to more demanding genomic regions and resulting in their depletion *in situ*.

Genome-wide studies in aging cells and tissues have uncovered epigenetic drift that reflects age-related decline of epigenetic integrity of the genome mediated by imperfect maintenance of epigenetic marks (for review, see [41, 42]). One important theme about key signature of aging chromatin or epigenetic drift is the loss of repressive marks (or heterochromatin) and gain of activating marks that have profound effects on gene expression [36]. In worms, knockdown of the H3K4me3 (activating mark) methyltransferase *set-2* extends lifespan while knockdown of the H3K4me3 demethylase *rbr-2* shortens lifespan [43], the two of which regulate H3K4me3 at global level [44]. In contrast, the expression of H3K27me3 (repressive mark) demethylase *utx-1* increases during worm aging, accompanied by a reduction of global H3K27me3 level [45]. RNAi depletion of *utx-1* in worm increases global levels of H3K27me3 and improves longevity [46], although in other aging model organisms the modulation of H3K27me3 methyltransferase or demethylase activity manifests a confounding phenotypes (for detail, see [36, 47]). In mammals, constitutive heterochromatin structure is disturbed in senescent cells, as evidenced by reduced global DNA methylation, H3K9me3, and HP1 levels [48, 49]. The loss of EZH2 and subsequent decrease of H3K27me3 level are marked in human senescent cells, and overexpression of EZH2 suppresses cellular senescence [35, 50, 51]. In human progeria models, global H3K9me3 (repressive mark) and H3K27me3 levels decrease [48, 52, 53]. We here showed in aged HD mice that H3K4 methyltransferases *Ash1l* and *Prdm9* were upregulated whereas *Ezh2* and H3K9 methyltransferases *Setdb1* were downregulated, and that H3K4 demethylases *Kdm1b* and *Kdm5c* were downregulated whereas H3K9 demethylase *Kdm4d* and H3K27 demethylase *Kdm6b* were upregulated (Fig. 5B). These orchestrated changes in the expression levels of epi-driver genes of certain categories predict a shift in chromatin to a more relaxed state which fits the general aging chromatin signature - heterochromatin loss.

Slight derepression of genes by epigenomic change in some portion of genome pool would result in many fold increase of expression compared to complete repression. On the other hand, the change in expression level would not be easily noticed from slight repression by epigenetic drift of actively expressed genes (it was, in reality, observed from the DNase-seq result in Fig. 6C). In HD mouse, the increase of expression level in the aged samples was broadly observed for the genes whose expression level was kept low or naught in young samples (Fig. 1E). Wt mice also exhibited this trend but far lesser degree. We therefore conclude that the tendency of epigenetic drift is more enhanced in HD than in wt mouse.

mHTT expression leads to reduced histone acetylation through its association with histone acetyltransferases, including CBP and P300/CBP-associated factor [17, 54], or aggregating CBP and other transcriptional regulators in the nucleus and cytoplasm [55]. Additionally, global histone hypoacetylation was induced in cells stably transfected with m*HTT* [54]. These studies reported mHTT toxicity to CBP and other histone acetyltransferases. In addition to the influence of mHTT at the protein level, we here analyzed the effect of mHTT on these histone acetyltransferases at the transcriptional level. We found that the amount of the acetylation-category transcripts in the aged samples by a similar level both in the wt and HD groups (the mean aged vs. young fold-changes were 0.665 (± 0.1406) and 0.780 (± 0.3292), respectively; Fig. 4). The result suggests that the reduced transcription of acetyltransferase genes was typical of aged cells, not further intensified in HD samples, and that mHTT is unlikely to regulate the histone acetyltransferases at the transcriptional level. Meanwhile, it is known that the sequestration of CBP protein by mHTT causes histone hypermethylation as well as hyperacetylation, and the subsequent transcriptional dysfunction of neurons in HD [56-60]. These observations were in agreement with our result of increased expressions (~1.4 fold) of K methylation-category genes specifically in aged HD samples, but not in the aged wt samples (Fig. 4). In HD brain, a loss of CBP leads to elevated expression of SETDB1, an H3K9 trimethylating enzyme [38, 39] and subsequently to condensation of heterochromatin structure [60, 61]. This is not the case with HD blood cells because *Setdb1* expression was oppositely slightly decreased in both aged wt and HD samples (Fig. 5B). This discrepancy probably reflects tissue-specific effects of mHTT.

The alteration in gene expression of the epi-driver genes in HD mice suggested a potential use of epigenetic drugs as therapeutics to mitigate the HD phenotype. Studies using models of polyglutamine diseases reported that HDAC inhibitors might serve as useful agents to ameliorate the polyglutamine pathology found in HD [6, 54, 55, 62, 63]. This is based on the hypothesis that if the polyglutamine pathology involves suppression of histone acetylation (or acetylation of other proteins), inhibition of the deacetylation process would slow or reduce polyglutamine pathogenesis *in vivo* [54]. Our results presented here showed that levels of deacetylation-category genes remained unchanged (*p* > 0.188) in the aged HD samples (Fig. 4), thereby supporting the possible use of HDAC inhibitors to increase histone acetylation in HD patients.

## MATERIALS AND METHODS

### HD model animals

In this study, we used YAC128 mice, which contain 128 CAG repeats of the human HTT gene under the control of yeast artificial chromosome [64], as HD model. These mice were previously reported with abnormal motor control and neuronal loss in striatum [64]. YAC128 mice were purchased from the Jackson Laboratories (Bar Harbor, ME, USA) and maintained under a 12 hours of dark/light life cycle in a temperature and humidity-controlled environment with free access to food and water. The presence of transgene in YAC128 was determined by genotyping with polymerase chain reaction (PCR) using primers as follows: forward 5′-CCGCTCAGGTTCTGCTTTTA-3′, reverse 5′-TGGAAGGACTTGAGGGACTC-3′. A total 7 of female littermate (wt) mice were used for this study at 16-19 months (*n*=4) and 2 months (*n*=3) of age. A total 7 of female mutant (HD) mice at 16-19 months (*n*=3) and 2 months (*n*=4) were used for this study. All procedures were performed with approval by Hanyang Institutional Animal Care and Use Committee (HY-IACUC-09-017).

### T cell isolation and nucleic acid preparation

Single-cell suspensions were prepared from spleens by mechanical dissociation, followed by removal of red blood cells (RBCs) with ACK Lysing Buffer (Lonza). T cells were further isolated by using Pan T cell Isolaton Kit II, mouse (Miltenyi Biotec). Untouched T cells were isolated with a mixture of CD11b, CD11c, CD19, CD45R, CD49b, CD105, Anti-MHC-class II, and Ter-119 and corresponding streptavidin microbeads on MACS-LS columns according to the manufacturer's protocol. Cell viability was assessed by trypan blue exclusion, and cell purity, which was > 95%, was determined by flow cytometry. Total RNAs were isolated from Magnetic-activated cell sorting (MACS) purified T-cells and macrophages using commercially available kit (RNeasy mini kit, Qiagen). cDNA was synthesized using iScriptTM cDNA Synthesis Kit (BioRad) according to the manufacture's instruction.

### Multiplex PCR

Mouse reference mRNA sequences for 260 genes were retrieved from the NCBI Reference Sequence Database and individually aligned to their respective rat genome sequence via BLAST to find homologous sequence stretches containing as few nucleotide inter-species variations (ISVs) as possible. The genes in this study were selected based on our own research interests: they are mostly related to epigenetic regulation mechanisms as we described elsewhere [34]. Homologous sequence stretches were subjected to Primer3 [65] to design primer pairs that bind both mouse and rat sequences simultaneously in PCR amplicon while encompassing the small numbers of ISV nucleotides in its amplicons to discriminate species origin. Primers were pooled into 19 groups of ~28 pairs in such a way that multiple primer pairs for a single transcript are separately assigned to different groups, to avoid the production of unintended amplicons. A multiplex PCR was carried out using each primer group with the following conditions: 15 min of enzyme activation at 95 °C followed by 45 cycles of 95 °C for 20 sec, 57 °C for 40 sec, and 68 °C for 1 min.

### Library construction and sequencing

All amplicons generated with 19 rounds of multiplex PCR were pooled together using equal volumes for each sample. For amplicon library construction, a series of enzymatic reactions with intermittent purifications using ExpinTM PCR (GeneAll) were performed, including 5′-end phosphorylation, adaptor ligation and two further PCR amplifications to attach the sequence module enabling flow cell attachment, sequencing primer binding and barcoding, as we described before [34, 66, 67]. The barcoded sequencing libraries were pooled with equivalent amount and subjected to a multiple parallel sequence using Illumina HiSeq 2500 platform.

### Species-specific reference sequences

Before identification of individual sequence reads, all sequences were processed to merge every pair of reads and to remove those with ambiguous nucleotide using the make.contigs command implemented in MOTHUR program [68]. Interspecific variations were initially inferred from the NCBI database used to construct temporary reference database (TEMP_REF) for BLAST analysis for the amplicon sequences derived from pure mouse and rat samples. The resulting alignment information was parsed using a series of Strawberry Perl (version 5.12.3.0) scripts. Reads that matched a reference sequence were collected and used to make consensus sequences. At each aligned position, sequences exhibiting at least 25% of total reads were retained. For each amplicon, consensus sequences form two species were compared and discard the amplicon if any consensus sequence of one species is identical to any of the other. A new BLAST reference database (ISV_REF) was created using the retained consensus sequences to consist of amplicon sequences with real ISVs.

### Quantitative analysis

The identity of all amplicon sequences from spike-in samples were determined by aligning them to ISV_REF reference database using BLAST. The read that matches to any of reference sequences with 100% sequence identity and a coverage of 90% were assigned to the reference sequence and counted. For each amplicon target, numbers of reads derived from the mouse mRNA and the rat genome were counted and their ratio was calculated. Deviations in the fractional quantities of amplicons from original values in the sample were corrected as previously reported [34]. The relative abundance of mouse to rat amplicon sequences (RA_M/R_) were then determined and normalized by dividing them with average relative abundance of all amplicons.

### RT PCR

Total RNA was prepared using RNeasy Plus Mini Kit (QIAGEN) according to the manufacturer's instructions, and cDNA was synthesized using Superscript III (Invitrogen). Primers used in PRG detection were as followings: 5′-ATAGTGGAAATGGCCCACCA-3′ and 5′-GGAGCTGACGGGAGATGAC-3′ for *Pax7*, 5′-ACCTTCTGGACAAGGACGTG-3′ and 5′-AGTGGCGCACGGAGCTA-3′ for *Cdx2*, 5′-TGGTCTTCACAGACGTCCAA-3′ and 5′-TTGGACGGACGCTTATTTTC-3′ for *Onecut1*, 5′-ATGGCCATGTACACGTTCTG-3′ and 5′-CTAGCGATATTTACAACGAA-3′ for *Nkx2.2*, 5′-AGTGTGAGTTCGAGGGCTGT-3′ and 5′-TGCATGTGCTTCTTCCTGTC-3′ for *Zic2*, and 5′-TCCACTCGTTCAGGAGGAAC-3′ and 5′-TGCTGTTATCCCCACTGTGT-3′ for *Meis1*. In order to detect mouse endogenous *Htt* expression, we used primers 5′-AGAGCCCCATTCATTGCC-3′ and 5′-TTCTTTGGTCGGTGCAGC-3 for *Htt* (1) and 5′-ACCCTGGAAAAGCTGATGA-3′ and 5′- TTCTTTGGTCGGTGCAGC-3′ for *Htt* (2). To detect human mutant HTT (mHTT) we used primers 5′-CCGCTCAGGTTCTGCTTTTA-3′ and 5′-TGGAAGGACTTGAGGGACTC-3′. PCR products were resolved on automatic electrophoresis machine (MultiNA, SHIMADZU). *Dnmt1* transcript level was measured with TOPreal™ qPCR 2x Premix (Enzynomics) using QuantStudio3 Real-Time PCR System (Applied Biosystems). Primers used for detection of *Dnmt1* transcript were 5′-CCACAGTGTTCACAGAGGA-3′ and 5′-CACACAGCATCTCCACATC-3′ [69].

### DNase-seq library generation

Five HD and six WT mice, aged fifteen to sixteen months, were sacrificed, and their spleens were removed for the isolation of mononuclear cells. Each spleen was ground, and cells were collected in cold DPBS (Gibco). Mononuclear cells (MCs) were isolated using Lymphoprep (STEMCELL) according to the manufacturer's protocol. After brief washing, number of isolated MCs were counted and aliquoted for the DNase I treatment. From 1x10^7^ HD or WT splenic MCs, nuclei were extracted using NE-PER Nuclear and Cytoplasmic Extraction Reagents (Thermo) according to the manufacturer's instruction, and the nuclei were digested with 5 units of DNase I (Takara) at 20 °C for 4 minutes. 100 μl Stop buffer (50 mM Tris-Cl, pH 8.0, 100 mM NaCl, 0.1% SDS, 100 mM EDTA, pH 8.0) was added to deactivate DNase I. The mixture was incubated with 2 μl RNase A (100 mg/ml, Thermo) at 55 °C for 10 minutes followed by 4 μl Proteinase K (10 mg/ml) for 2 hours to free the DNAs from proteins. DNAs were purified using phenol/chloroform and ethanol precipitation methods, and 50 ~ 100 bp DNA fragments were size-selected for Illumina NGS library preparation. The size selected DNA fragments were used for Illumina NGS libraries using TruSeq DNA Sample Prep Kit (Illumina) according to the manufacturer's instruction. The resulting NGS libraries were sequenced by HiSeq2000 (Illumina).

### DNase-seq data analysis

Raw sequencing reads were pre-processed to removed unwanted bases, and the resulting reads were mapped on a mouse reference genome (mm10) using ‘bowtie2’ [70] with default options. Mapping efficiency was assessed by visualizing the mapped reads on IGV. To calculate read density around TSS and draw heatmaps, ‘deepTools’ [71] was used. All other plots were generated by in-house R scripts.

## SUPPLEMENTARY MATERIALS FIGURES


