# Supplementary material for: Age-associated chromatin relaxation is enhanced in Huntington's disease mice
Source: Aging (Albany NY). 2017 Mar 12;9(3):803–18. doi: 10.18632/aging.101193 (PMC5391233; doi:10.18632/aging.101193)
Supplement: Supplementary file 1 [file aging-09-803-s001.pdf]

SUPPLEMENTARY MATERIALS

Supplemental Figures.

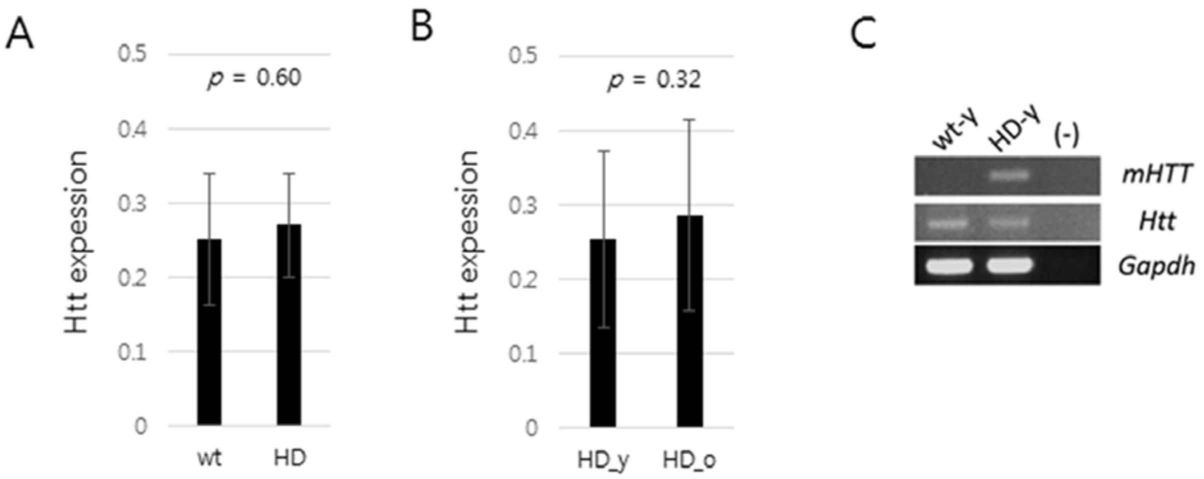

Figure S1. Huntingtin (*Htt*) expression in wild-type (wt) and mouse models of Huntington's disease (HD). (**A**) *Htt* expression levels in splenic T cells from wt and HD mice. Average expression levels of young and aged mice were calculated. (**B**) *Htt* levels in young (HD_y) and aged (HD_o) HD mice. (**C**) Detection of human mutant *HTT* (*mHTT*) and endogenous *Htt* gene expression in HD mice. (−), no template control.

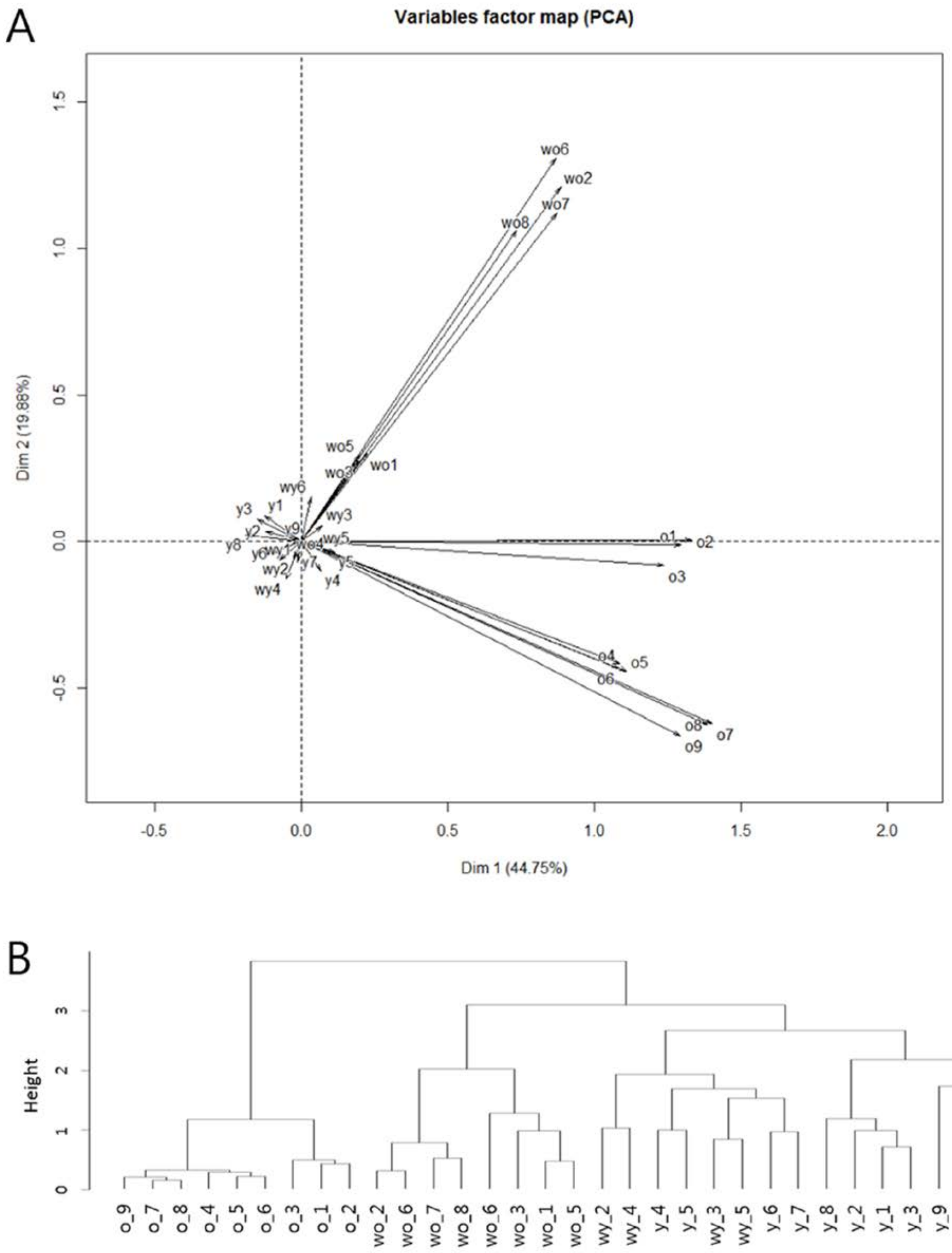

Figure S2. Correlation analysis. (**A**) Principal component analysis (PCA). Four groups are included: wild-type young (wy), wild-type old (wo), Huntington's disease (HD) young (y), and HD old (o). (**B**) Unsupervised cluster analysis.

Gene id	Category	No. Count	M/R ratio* in young	M/R ratio* in aged	log_2FC	P-val	FDR
**Ig1_55**	Ageing	510	0.004	0.392	6.515	1.39E-16	4.41E-14
**Clu_2**	Ageing	13224	0.046	1.457	4.980	1.4E-12	2.23E-10
**PADI4_1**	Arg_meth	3139	0.002	0.205	6.530	4.02E-12	4.26E-10
**Clu_1**	Ageing	1268	0.041	0.636	3.951	5.89E-08	0.00000468
**PRMT6_1**	Arg_meth	919	0.041	0.591	3.847	0.000000116	0.00000739
**Cd44_1**	Ageing	2140	0.010	0.195	4.248	0.00000057	0.0000302
**Casp1_2**	Ageing	5674	0.353	2.104	2.577	0.000000977	0.0000444
**RPRD1A_2**	etc	5537	0.066	0.821	3.644	0.00000123	0.000049
**Casp1_1**	Ageing	14597	0.870	5.325	2.613	0.00000431	0.000152169
**RNF38_5**	Ubiq	2148	0.109	1.130	3.378	0.0000193	0.000612567
**Il6_1**	Ageing	1428	0.007	0.131	4.138	0.0000591	0.001708404
**JMJD7_1**	Lys_demeth	3779	0.323	1.534	2.246	0.000338713	0.008975895
**Rel_26**	Ageing	1733	0.145	0.944	2.704	0.000437244	0.009931695
**Ier3_1**	Ageing	463	0.129	0.855	2.724	0.000426953	0.009931695

* M/R ratio, the ratio of the mouse sequence counts relative to the rat's counts

Figure S3. Differentially expressed target sequences between wild-type young and aged mice. Gene id, gene symbol_amplicon number. Expression level of each amplicon was measured by calculating the ratio (M/R) of cDNA counts relative to rat spike-in counts. No Count, the number of read count of each amplicon. FC, fold change. FDR, false discovery rate.

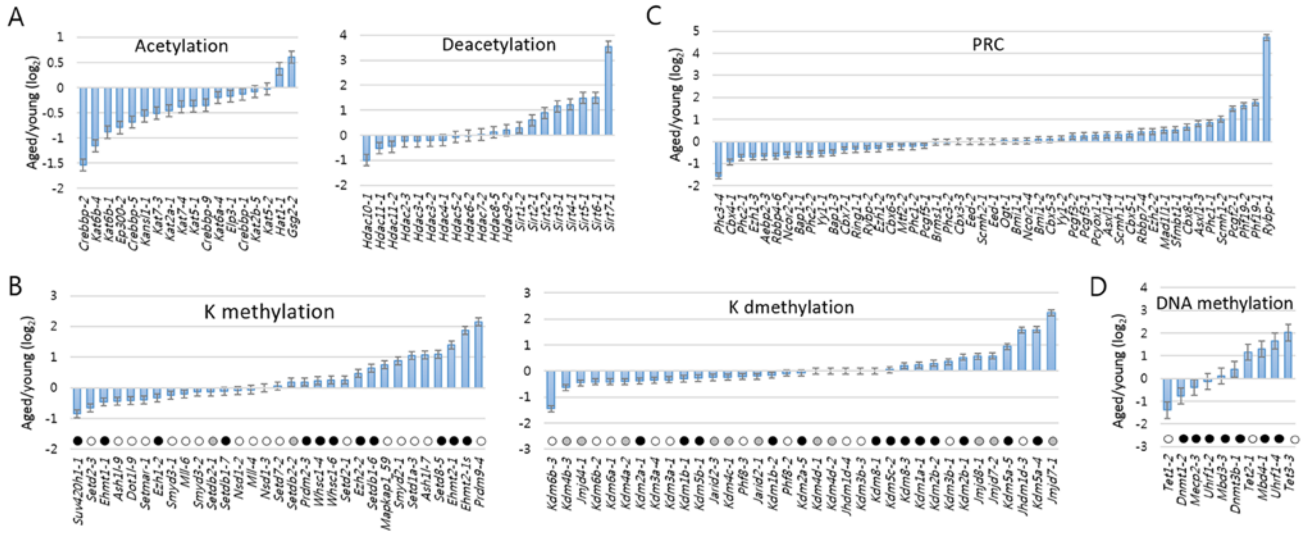

Figure S4. Fold changes in the amount of amplicons in aged wild type mice compared to young mice. Fold changes were measured for the epi-driver gene amplicons in the categories of acetylation and deacetylation (**A**), lysine (K) methylation and K demethylation (**B**), Polycomb-repressive complex (PRC) (**C**), and DNA methylation (**D**). In B and D, amplicons are differentially marked according to the modification effects of their proteins on chromatin accessibility: open circles indicate increased accessibility; solid circles indicate reduced accessibility; and grey circles for cases involving either increased or reduced accessibility.

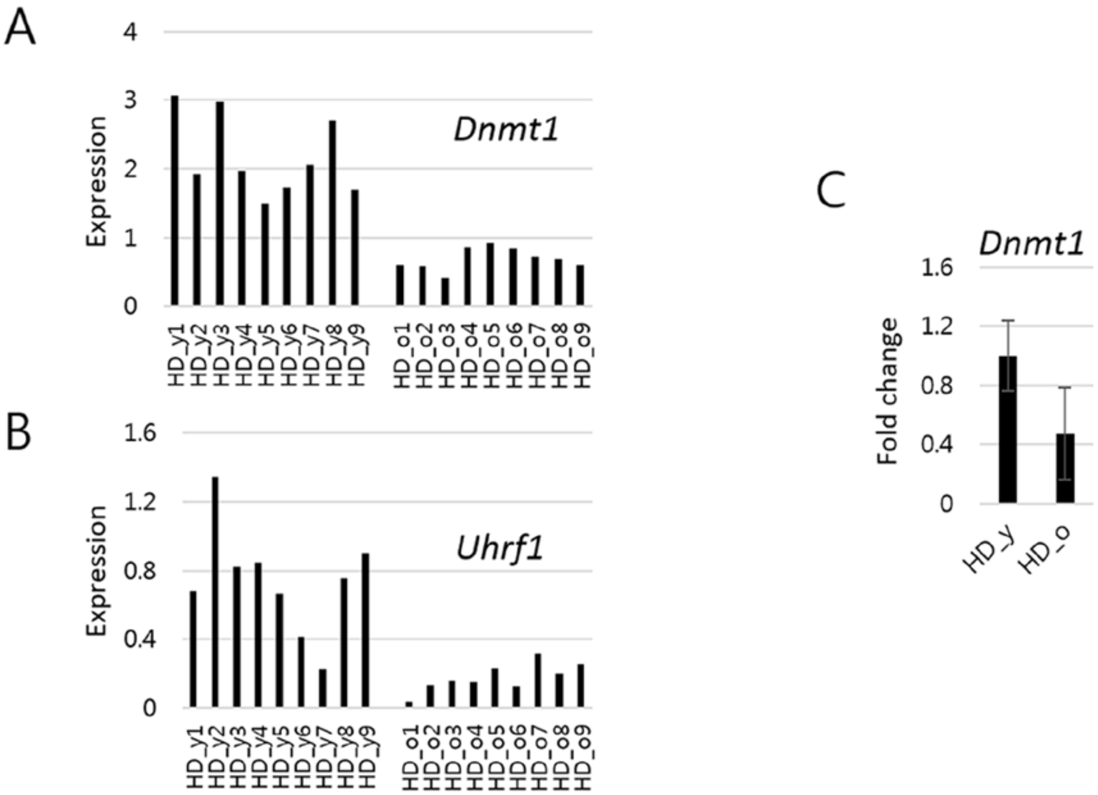

Figure S5. Amplicon levels of *Dnmt1* (**A**) and *Uhrf1* (**B**) in young and aged mouse models of Huntington's disease (HD_y and HD_o, respectively). (**C**) quantitative real-time PCR analysis of *Dnmt1* gene expression.
